# The role of circulating T cells with a tissue resident phenotype (ex-T_RM_) in health and disease

**DOI:** 10.3389/fimmu.2024.1415914

**Published:** 2024-05-16

**Authors:** Beverley Rodger, Andrew J. Stagg, James O. Lindsay

**Affiliations:** ^1^ Blizard Institute, Barts and The London Faculty of Medicine and Dentistry, Queen Mary University of London, London, United Kingdom; ^2^ Department of Gastroenterology, Royal London Hospital, Barts Health NHS Trust, London, United Kingdom

**Keywords:** tissue-resident memory T cells, ex-T_RM_, tissue egress, recirculation, inflammatory disease

## Abstract

Tissue-resident memory T cells (T_RM_) are long-lived memory lymphocytes that persist in non-lymphoid tissues and provide the first line of defence against invading pathogens. They adapt to their environment in a tissue-specific manner, exerting effective pathogen control through a diverse T cell receptor (TCR) repertoire and the expression of proinflammatory cytokines and cytolytic proteins. More recently, several studies have indicated that T_RM_ can egress from the tissue into the blood as so-called “ex-T_RM_”, or “circulating cells with a T_RM_ phenotype”. The numerically small ex-T_RM_ population can re-differentiate in the circulation, giving rise to new memory and effector T cells. Following their egress, ex-T_RM_ in the blood and secondary lymphoid organs can be identified based on their continued expression of the residency marker CD103, alongside other T_RM_-like features. Currently, it is unclear whether exit is a stochastic process, or is actively triggered in response to unknown factors. Also, it is not known whether a subset or all T_RM_ are able to egress. Ex-T_RM_ may be beneficial in health, as mobilisation of specialised T_RM_ and their recruitment to both their site of origin as well as distant tissues results in an efficient distribution of the immune response. However, there is emerging evidence of a pathogenic role for ex-T_RM,_ with a suggestion that they may perpetuate both local and distant tissue inflammation. Here, we review the evidence for the existence of ex-T_RM_ and examine their potential involvement in disease pathogenesis.

## 1 Introduction

**Figure 1:**
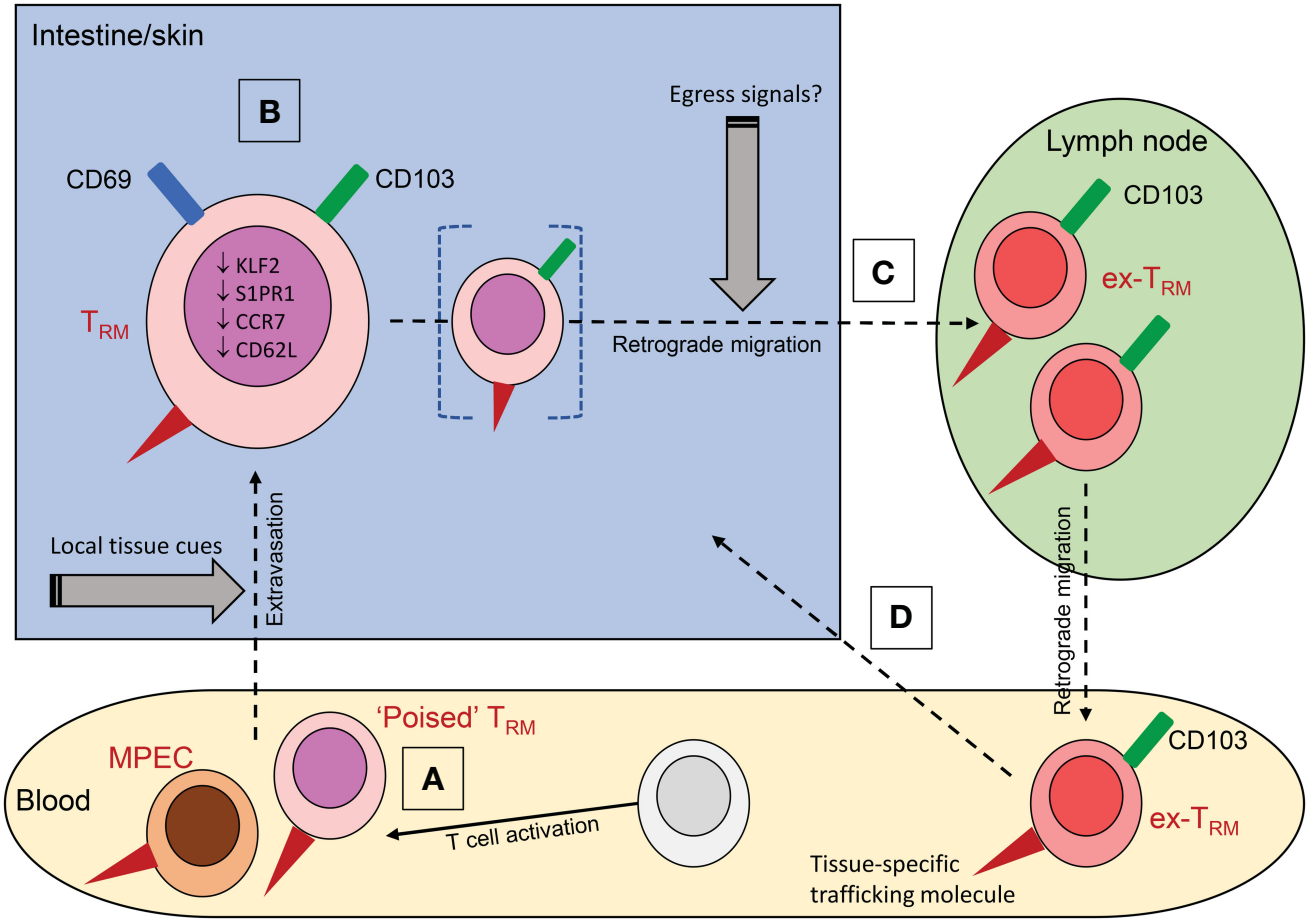
The origins and properties of ex-T_RM_. **(A)** Memory precursor cells (MPEC) recruited into tissues can differentiate into T_RM_ under the influence of local tissue cues including cytokines; a subset of these circulating precursors may be pre-committed to a T_RM_ fate (‘poised T_RM_’). **(B)** Residence is associated with expression of CD69 and CD103 and downregulation of the transcriptional profile associated with tissue egress. **(C)** Under conditions that are poorly defined, T_RM_ can downregulate CD69, while retaining CD103 expression and undergo retrograde migration to the draining lymph node or beyond into the blood. The ability to egress may be a property of a distinct subpopulation of cells (bracketed in figure). Circulating ex-T_RM_ are plastic cells that can undergo further differentiation to yield a variety of effector and memory T cells. **(D)** They can re-enter tissue, with a propensity to return to the tissue of origin due to retained expression of tissue-specific trafficking molecules (red triangles in figure); these include CLA and α4β7 for skin and intestine respectively.

T cells are crucial for the generation of a successful adaptive immune response against pathogens. Upon entry into barrier tissues, antigen presenting cells (APCs) capture antigen from the invading pathogen and traffic it to the local draining lymph node, where naïve antigen-specific T cells are then activated (1). This is followed by a period of rapid clonal expansion and differentiation, following which newly generated effector T cells migrate to the site of infection and participate in host defence through their production of cytokines and direct cytolytic activity (2–4). Most effector T cells die following pathogen clearance, but a small fraction survives and is capable of recirculating between non-lymphoid tissue and the blood (5). Referred to as memory precursor effector cells (MPECs), this population can differentiate into memory T cells, thus ensuring a potent recall response against invading pathogens (2). Memory T cells can be broadly divided into circulating and resident memory populations, the latter of which was identified a little over a decade ago (2, 6–8). The circulating memory population comprises central memory T cells (T_CM_), which proliferate extensively upon reactivation, giving rise to expanded effector cell populations, and terminally differentiated effector memory T cells (T_EM_) which rapidly differentiate into new effector cells upon antigen re-encounter (2, 9). The tissue memory compartment comprises resident memory T cells (T_RM_), which are localised in non-lymphoid tissues and serve as a “frontline” defence against recurring pathogens at barrier sites (6–8, 10). T_RM_ are phenotypically and transcriptionally distinct from other T cell populations (11) but share features with circulating T_EM_ and effector T cells, including their expression of proinflammatory cytokines and cytolytic proteins, meaning they are primed to rapidly respond to infection. They can proliferate *in situ*, driving autonomous expansion of their population (10, 12). To date, studies on T_RM_ have focused mostly on CD8^+^ cells in the mouse, but the importance of CD4^+^ T_RM_ is increasingly recognized (13) and equivalent populations have been identified in humans. Until recently it was assumed that once residency is established T_RM_ do not exit the tissue. However, several recent studies have suggested that a fraction of T_RM_ are capable of egress and can be found in draining secondary lymphoid organs (SLOs) and in the circulation (**Figure 1**) (14–19). Here, we will review the evidence for the existence of ex-T_RM_, discuss their properties, and highlight the emerging evidence for their potential involvement in inflammatory disease.

## 2 The origin and differentiation of T_RM_

Cells with the phenotype of T_RM_ are present in most, if not all, human tissues (20, 21), and studies in transplant patients have directly demonstrated long term maintenance of donor-derived T cells in the transplanted tissue (22–24). Despite their prevalence, the origin and development of T_RM_ is not fully understood. T_RM_ are present in both the human fetal and infant intestine, suggesting T_RM_ seeding and development can occur early in life in response to ingested antigens, but the precise nature of the precursor population giving rise to these cells is unclear (25–27).

T_RM_ differentiation is induced by exposure to tissue-derived factors following extravasation of precursors (5, 27, 28). It can be influenced by pre-commitment to this fate prior to arrival in the tissue (28); T cells clones have been identified within circulating mouse T cells that already express T_RM_ associated genes and have an enhanced potential to establish residency in the skin (28). This pre-commitment to a T_RM_ fate occurs early in an immune response and it can occur either within the naïve T cell population (29) or following activation through the TCR, possibly as the result of interaction with specific DC populations (5, 28–32). Regardless of their origin, T_RM_ precursor cells are thought to give rise to T_RM_ by an induced differentiation programme (19, 33–36). The precise signals resulting in the acquisition of a T_RM_ phenotype are complex and likely to be tissue-specific, but there is evidence to suggest that interaction with the extracellular matrix, cytokines including IL-15 and TGFβ, hypoxia, and persistent antigenic stimuli are important for T_RM_ generation in certain contexts (34–38). In response, a number of transcription factors including Runx3, Notch, Hobit, and Blimp-1, function together as a module to silence lymphocyte egress pathways (39–41). These positive, tissue-derived developmental cues result in phenotypic, transcriptional, and metabolic changes to the cell enabling it to attain residency within the tissue.

The conversion of T_RM_ precursor cells to T_RM_ is coupled with the upregulation of tissue retention molecules, including CD69 and CD103 (α_E_β7 integrin). CD69 promotes residency by triggering internalisation of sphingosine-1-phosphate receptor 1 (S1PR1) which otherwise mediates migration from the tissue in response to S1P gradients, whereas CD103 binds to E-cadherin and tethers cells to the epithelium (11, 42, 43). T_RM_ are also characterised by reduced expression of the transcription factor KLF2, which promotes *S1PR1* expression and a number of additional cell surface markers linked to lymph node-homing and tissue-egress molecules, such as CCR7 and CD62L (11, 44, 45). CD69 and CD103 are often used as phenotypic markers of T_RM_. CD103 is typically expressed by a subset of CD69^+^ cells, constituting a greater proportion of CD8^+^ than of CD4^+^ T_RM._ CD103^+^ and CD103^-^ T_RM_ subsets have distinct functions during infection (46, 47). It should be emphasized that the biology of T_RM_ is complex and caution should be exercised when extrapolating findings to different cell subsets, tissues, or species.

Once resident within the tissue, T_RM_ respond rapidly to infection owing to their diverse tissue-specific TCR repertoire, and close proximity to the cellular targets of specific pathogens (8, 48–52). Upon re-activation, they display immediate cytotoxic potential, and can rapidly release proinflammatory cytokines such as such as IFNγ and TNFα (53, 54). The cytokines released by T_RM_ can render surrounding cells resistant to infection, even with unrelated antigens, and can create tissue-wide anti-pathogen responses via the upregulation of Type I IFN signalling pathway factors and the enhanced recruitment of leucocytes (53, 54). In addition, T_RM_ are able to kill infected cells directly via production of the cytolytic proteins perforin and granzyme B, enabling them to induce the contact-mediated apoptosis of infected cells they encounter (55, 56).

## 3 Experimental evidence for the existence of ex-T_RM_

For years T_RM_ were thought to be permanently resident within the tissue without recirculating. However, it has recently been observed that some T_RM_ are capable of egressing into draining SLOs (57, 58) or the blood (14, 15, 18, 19), a pattern of trafficking that has been termed retrograde migration (15, 58). The cells in the blood, herein termed ex-T_RM,_ can be identified by their continued expression of CD103 and other T_RM_-like phenotypic features (14, 19, 59). They are plastic cells that contribute to the circulating memory response by re-differentiating into other memory T cell subsets and can enter tissue to re-establish residency (14, 15, 17, 19). In a key study, a fraction of the CD4^+^CD103^+^ T_RM_ population present in human skin was found to be capable of downregulating CD69 expression and migrating from the tissue. In an allograft model these cells re-entered the circulation, and migrated to a secondary skin site where they subsequently reassumed their T_RM_ phenotype (14). A corresponding population was detected at low frequency in human blood based on continued expression of CD103 in conjunction with the skin-specific trafficking molecule CLA (cutaneous leucocyte antigen). These cells shared a transcriptional and phenotypic profile with the skin resident population, strengthening the argument that they were related to a *bona fide* resident population. Importantly, TCRβ sequencing confirmed that both circulating CD4^+^CD103^+^ cells and T_RM_ from different skin sites were clonally related, thus highlighting their ability to seed distant tissue locations (14). The continued expression of CLA by the skin derived ex-T_RM_ in the circulation enables them to home preferentially back to their tissue of origin (14). These cells may be related to recirculating T cells previously described in human skin (60). Recent studies of patients undergoing hematopoietic stem cell transplant (HSCT) have also provided evidence for the existence of skin derived ex-T_RM_ (16). In this context the circulating T cell pool is rapidly replaced with donor T cells, but host-derived skin T_RM_ survive conditioning and are maintained for at least ten years, during which time they can egress and be found in the circulation (16, 61). The recipient-derived circulating T cells were identified transcriptionally based on their high expression of *ITGAE* (encoding CD103) and *SEPLG* (the gene encoding the protein backbone of CLA). The data discussed above on healthy human skin would suggest that this is not a phenomenon restricted to the unique situation of HSCT.

A mouse study supports the concept that T_RM_ can also exit the intestine and has highlighted the plasticity of T_RM_ during this process. Murine intestinal T_RM_ were shown to undergo retrograde migration (15) and whilst circulating, the ex-T_RM_ population exhibited developmental plasticity and differentiated into T_CM_ or T_EM_ in the circulating pool (15). Interestingly, the circulatory intestinal ex-T_RM_ retained a preference to home back to their tissue of origin, in this case the intestine, and reacquired a stable T_RM_ differentiation programme in response to local cytokine cues (15, 19). The preference of ex-T_RM_ to return to their original tissue was seen even following tertiary reinfection with LCMV, suggesting the maintenance of a stable T_RM_ profile over time (15). Although the phenomenon of ex-T_RM_ is a relatively recent discovery, the presence of these cells may have first been reported several years ago. Jiang et al. (55) first described a population of vaccinia virus-specific CD8^+^ T_RM_ in mice that could be found at skin sites distant to the site of initial infection. This remote population was highly effective at rapidly eliminating virus and was able to reduce viral loads to those seen at primary sites of infection (55). At the time it was believed this population derived from circulating T_EM_ cells that randomly distributed into skin sites where they differentiated into T_RM_ (55). However, this may be one of the first reports of skin ex-T_RM_ egressing from the initial site of infection and seeding secondary sites to enhance local immunity. This phenomenon was more recently reported following the use of a murine human skin xenograft model (14).

## 4 Ex-T_RM_ in health and disease

**Figure 2:**
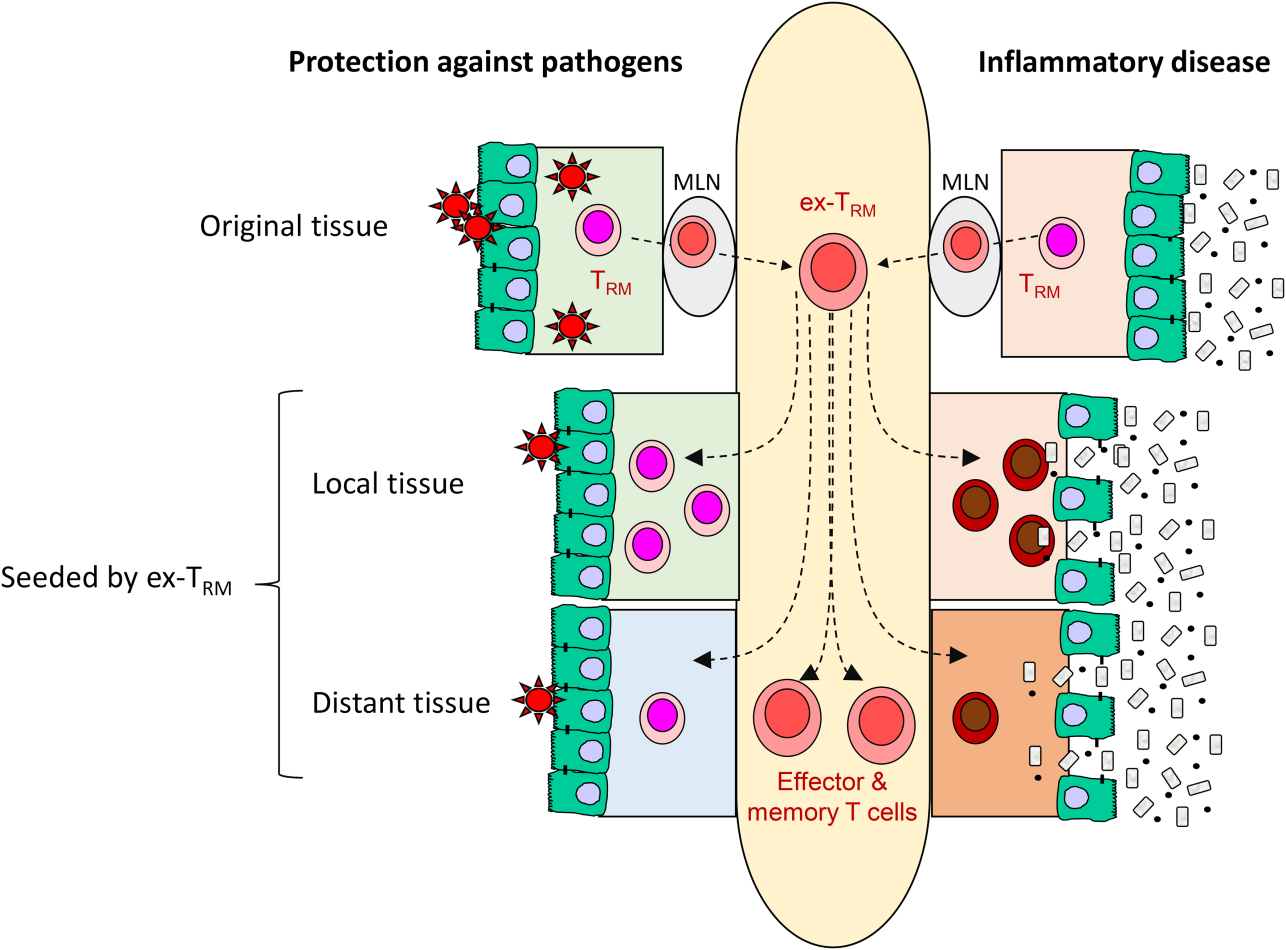
The potential contribution of ex-T_RM_ to protective immunity and inflammatory disease. Here, potentially beneficial and detrimental contributions of ex-T_RM_ are illustrated with refence to the intestine. The plasticity of ex-T_RM_ enables them to generate a variety of effector and memory cells T cells as well as re-establish tissue residency. In the context of infection, these properties may form part of an early warning system responsive in initial stages of colonization (‘outside in’ immunity), provide cellular reinforcements to the site of infection and provide enhanced protection at remote sites that might be vulnerable to the pathogen (left side of figure). However, in chronic infmmatory disease, the same properties may enhance the local deleterious response and spread the inflammation to distant sites within the same tissue or even to other organ systems (right side of figure). MLN: mesenteric lymph node.

There is considerable evidence that the egress of T_RM_ from tissue occurs in health, generating TCR clonotypes shared between barrier sites, such as the skin or intestine, and the circulation (62, 63). In this context, the ability of T_RM_ to redistribute and contribute to the circulating memory pool is likely to be beneficial. This ‘outside-in’ immune response (15) brings several benefits to the host in the context of infection (**Figure 2**). Firstly, should an infection overwhelm the local T_RM_ response, there are cells within the circulating compartment which retain a preference to home back to the infected tissue and can re-establish local immunity (15). Secondly, in the case of a large barrier organ such as the gut or skin, infection could occur at any location within the organ and not necessarily at a previous site of infection (19). Thus, the ability of specialised T_RM_ to be mobilised from one site and recruited back from the circulation to other sites results in an efficient distribution of the immune response (19).

However, the presence of ex-T_RM_ may be detrimental in the setting of an immune-mediated disease, and it is conceivable that the redistribution of potentially pathogenic T_RM_ populations may perpetuate both local and distant tissue inflammation. There are currently limited data about ex-T_RM_ in immunological disease but in HSCT, the recipient skin-derived ex-T_RM_ population discussed above induce keratinocyte cell death and tissue damage through their expression of Th2 and Th17-associated cytokines such as IL-13 and IL-17 (16). In addition to skin trafficking molecules, they expressed *ITGA4* and *ITGB7*, encoding the integrin α4β7 which facilitates entry to the intestine, and were localised in gastrointestinal graft versus host disease (GVHD) lesions. These findings not only highlight a potentially pathogenic role for ex-T_RM_, but also suggest their involvement in the propagation of inflammation to distant organs (16, 61). Potential evidence linking the aberrant trafficking of ex-T_RM_ to distant tissue inflammation has also been found in ankylosing spondylarthritis (AS), a disease characterised by chronic inflammation of the axial skeleton (62). Proteomics and transcriptomic analysis revealed the presence of a specific population of mature CD8^+^ T cells elevated in both the blood and synovial fluid of AS patients, which were enriched for the cell-surface expression of gut-associated trafficking molecules, including β7 integrin, CD103, and CD49a (64). These cells also exhibited a dual cytotoxic and regulatory profile, with the release of TNFα, perforin, and IL-10 (64). Bulk RNA-sequencing of the CD8^+^ T cell population revealed a core transcriptional signature reminiscent of T_RM_, with the upregulation of residency-associated molecules *ITGAE* (CD103) and *CXCR6*, and a downregulation of tissue egress factors including *SELL* (CD62L) and *S1PR1* (64). The expression pattern of these molecules was similar to that of intestinal intraepithelial lymphocytes (IELs), leading the authors to hypothesise that the CD8^+^ T cell population in the blood and synovial fluid may have mucosal origin, potentially having been trafficked from the gut to the joint (64). Interestingly, AS often co-exists with inflammatory bowel disease (IBD), a disease with which it shares many clinical, genetic, and immunological overlaps (64, 65). Given the gut-homing integrin α4β7 is also expressed by immune cells in the synovial tissue, a potential explanation for the co-existence of these conditions may be that inflammation in the gut is transferred to the joint through the aberrant trafficking of ex-T_RM_ cells (64).

There is also recent evidence to suggest that the frequency of ex-T_RM_ may be increased in inflammation, possibly indicating a role for inflammatory signals in the egress of T_RM_ from tissue. In inflammatory bowel disease (IBD; Crohn’s disease and ulcerative colitis), intestinal T_RM_ have been implicated in the inflammatory process (66). Single-cell RNA sequencing (scRNA-seq) analysis of blood and intestinal tissue from ulcerative colitis patients revealed a marked increase in TCR clonotypes shared between a CD8^+^ T cell cluster derived from intestinal tissue and a cluster derived from peripheral blood, suggesting a migration of clonally related cells between the two compartments (62). Interestingly, the proportion of shared clonotypes was significantly higher in ulcerative colitis patients compared with healthy controls; potentially indicating more frequent or increased T_RM_ egress in a disease setting (62). Further, the presence of a small subset of peripheral blood CD4^+^ T cells with similar characteristics to synovial CD4^+^ T cells is correlated with both disease activity and resistance to therapy in juvenile idiopathic arthritis (JIA) (67). Both blood and synovial CD4^+^ populations exhibited a high degree of clonal overlap, alongside phenotypic similarities including increased IL-17, IFNγ, and TNFα production, thus suggesting the retention of an active T_RM_ proinflammatory profile in the circulation (67). The host-derived ex-T_RM_ population is also increased in the blood of HSCT patients with active GVHD, potentially providing a further link between inflammatory factors and T_RM_ egress (16).

Much of the limited data currently available on the role of ex-T_RM_ in disease is associated with skin and gut conditions, perhaps reflecting the large size of these organs and their greater contribution to the ex-T_RM_ pool. Ex-T_RM_ may also contribute to other autoimmune and inflammatory disease, but this possibility largely remains unexplored.

## 5 Discussion

Whilst the above-mentioned recent work has identified populations of ex-T_RM_ in both murine and human peripheral blood, we are still left with a number of unanswered questions. Firstly, although there is strong evidence that these cells are tissue-derived and share many features with the resident population, the possibility that they are derived from cells distinct from the fully committed T_RM_ population (35) has not been definitively excluded. Further fate mapping in mice or analysis of ex-T_RM_ derived from transplanted human tissue may help resolve this issue. Secondly, it is still unclear whether only a small proportion of the T_RM_ population can egress, or whether all T_RM_ are capable of re-entering the circulation. Given the rarity of ex-T_RM_ in peripheral blood - where they represent less than 1% of the total PBMC compartment – it is perhaps likely that only a small proportion of the vast T_RM_ population are equipped to egress as ex-T_RM_ (16). The ex-T_RM_ so-far identified in blood are from skin and intestine and this may reflect the size of these organs, with the large number of immune cells they contain making rare exit events detectable. Analysis following separation of parabiotic mice show a gradual accumulation of blood ex-T_RM_ over time (18). It is possible that retrograde migration as far as SLOs is a relatively common event but further progress into the circulation occurs only infrequently. Alternatively, the small number of ex-T_RM_ identified in peripheral blood by phenotyping approaches may be explained by a change in phenotype following exit from the tissue. CD103 expression, alongside that of tissue-specific homing markers, is currently the best-defined method by which to identify ex-T_RM_ populations (14–16). Therefore, should ex-T_RM_ rapidly downregulate CD103 following their egress from tissue, they would go largely undetected with significant numbers of cells being missed. Future work should thus aim to identify additional transcriptional or phenotypic characteristics of this population. Finally, studies are also required to address whether the mobilization of resident cells is a stochastic process, or whether it is triggered by as-yet undefined factors. It is possible that there is a consistently low level of T_RM_ egress when the tissue is at steady-state, but this may increase in the presence of inflammation, potentially as a result of increased signaling or antigen re-encounter. In some experimental systems egress of CD103^+^ T_RM_ has not been seen to occur (47), perhaps suggesting that particular exit signals are lacking in these contexts. Given the potential involvement of ex-T_RM_ in disease pathogenesis, it is important that future studies aim to address these pertinent questions in a range of conditions.

## 6 Concluding remarks

Recent experimental evidence in mice and humans suggests a fraction of T_RM_ are capable of egressing from the tissue to become ex-T_RM_ which are able support protective immune responses but may also be able to perpetuate local and distant tissue inflammation. Understanding the mechanisms underlying the generation and egress of ex-T_RM_ may provide new therapeutic targets for disease.

## Author contributions

BR: Writing – original draft, Writing – review & editing. AS: Conceptualization, Funding acquisition, Supervision, Writing – original draft, Writing – review & editing. JL: Conceptualization, Funding acquisition, Supervision, Writing – original draft, Writing – review & editing.
